# Fetal Aberrant Right Subclavian Artery: Associated Anomalies, Genetic Etiology, and Postnatal Outcomes in a Retrospective Cohort Study

**DOI:** 10.3389/fped.2022.895562

**Published:** 2022-06-03

**Authors:** Meiying Cai, Na Lin, Xiangqun Fan, Xuemei Chen, Shiyi Xu, Xianguo Fu, Liangpu Xu, Hailong Huang

**Affiliations:** ^1^Medical Genetic Diagnosis and Therapy Center, Fujian Maternity and Child Health Hospital, College of Clinical Medicine for Obstetrics & Gynecology and Pediatrics, Fujian Medical University, Fujian Key Laboratory for Prenatal Diagnosis and Birth Defect, Fuzhou, China; ^2^Guangxi Medical University, Guangxi, China; ^3^Department of Prenatal Diagnosis, Ningde Municipal Hospital, Ningde Normal University, Ningde, China

**Keywords:** aberrant right subclavian artery (ARSA), single-nucleotide polymorphism, copy number variation, ultrasound abnormalities, postnatal outcomes

## Abstract

**Background:**

Aberrant right subclavian artery (ARSA) is becoming increasingly common in fetuses. However, there are relatively fewer studies regarding the genetic etiology of ARSA. We performed a genetic analysis of fetuses with ARSA and followed up on the pregnancy outcomes to evaluate the prognosis of the fetuses, providing information for prenatal and eugenic consultations.

**Methods:**

This retrospective study included 112 pregnant females whose fetuses were diagnosed with ARSA from December 2016 to February 2021. Fetal karyotype analysis and single-nucleotide polymorphism (SNP) array were performed.

**Results:**

The 112 fetuses were divided into two groups: the isolated ARSA group (*n* = 48, 42.9%) and the non-isolated ARSA group (ARSA with other ultrasound abnormalities, *n* = 64, 57.1%). The total rate of pathogenic copy number variation (CNV) observed using karyotype analysis (3/8) and SNP array (5/8) was 7.1% (8/112). The rates of pathogenic CNV in the isolated and non-isolated ARSA groups were 4.2% (2/48) and 9.4% (6/64), respectively. No significant difference was observed between the two groups (*P* = 0.463). The results of genetic analysis influenced the parents’ decision to terminate the pregnancy. During the follow-up examination, fetuses with ARSA without pathogenic CNV were found to have normal growth and development after birth.

**Conclusion:**

Fetuses with isolated ARSA have a low probability of being diagnosed with pathogenic CNV. However, when ARSA is complicated with other ultrasound abnormalities, the risk of pathogenic CNV remarkably increases. Prenatal genetic counseling and SNP-array should be recommended for better assessment of fetal prognosis.

## Introduction

Aberrant right subclavian artery (ARSA) is a common congenital anomaly of the aortic arch, observed in 0.5–1.4% of healthy individuals (1–3). ARSA can either be a normal vascular variation or a part of a complex heart malformation or genetic syndrome. Normal ARSA arises from the aortic arch, one of the branches of the cephalic arm trunk, and is the first branch of the aortic arch. ARSA originates at the beginning of the descending aorta, and after diverging from the left subclavian artery, the fourth branch of the aortic arch passes close to the trachea and goes to the right shoulder (4).

In recent years, owing to the advances in prenatal ultrasound diagnosis techniques employed in maternal and fetal medicine, increasing number of fetal structural malformations are being diagnosed. This can help clinicians better formulate an effective diagnosis and treatment plans, reduce adverse pregnancy outcomes, and enhance the prevention and control of birth defects (5). The C-type vascular ring formed by ARSA is an incomplete vascular ring, which partially surrounds the trachea and esophagus, typically without compressing them (6). Generally, ARSA does not demonstrate any apparent clinical symptoms; however, in rare cases, it can compress the esophagus or trachea, causing difficulties in swallowing or breathing (7). Prenatal ultrasound can accurately diagnose fetal ARSA. Fetuses with ARSA are at an increased risk of trisomy 21 syndrome and copy number variation (CNV) (8–11). Ultrasonographic soft markers indicate abnormalities other than the anomalous ultrasonic structures observed during the ultrasound examination. Such markers are non-specific indices and do not accurately indicate the structural abnormality of the fetus. Furthermore, they may also be regarded as normal variations. ARSA can be used as an ultrasound soft marker for prenatal screening of the fetal chromosomal abnormalities (12).

In this study, fetuses with ARSA diagnosed by the prenatal ultrasound were included, and genetic etiology of fetuses with ARSA was detected by karyotype analysis and single-nucleotide polymorphism (SNP) array. This study aimed to analyze the correlation between ARSA and genetic etiology, postnatal outcomes, and prognosis and provide information for prenatal and eugenic consultations.

## Materials and Methods

### Participants

A retrospective study was conducted with 112 pregnant females, whose intrauterine fetuses were diagnosed with ARSA during ultrasound examination at the Fujian Maternal and Child Health Hospital from December 2016 to February 2021. The age of the participants ranged from 18 to 46 years, with an average of 28 ± 5 years. The gestational age ranged from 17 to 35 weeks, with an average of 24.6 weeks. After the participants and their family members signed the informed consent form, amniotic fluid or cord blood was collected for karyotype analysis and SNP-array according to different gestational weeks. The 112 cases were divided into the isolated ARSA group (48 cases) and non-isolated ARSA (ARSA combined with other ultrasonic abnormalities) group (64 cases). This study was approved by the Ethics Committee of Fujian Maternal and Child Health Hospital (no. 2014-042), and the subjects signed the informed consent forms. We confirm that all the experiments were performed in accordance with relevant guidelines and regulations.

### Karyotype Analysis

The amniotic fluid or cord blood samples of 112 fetuses with ARSA were cultured and g-banded according to the conventional methods established a tour center (13). Karyotype was collected and analyzed by GSL-120 automatic chromosome scanning platform. In total, 20 karyotypes were counted in each case, 5 were analyzed, and the count and analysis were increased in the case of abnormality.

### Single-Nucleotide Polymorphism-Array

Genomic DNA was extracted from fetal cells using QIAamp DNA Blood Mini Kit. Digestion, amplification, purification, fragmentation, labeling, hybridization with microchips, washing, scanning, and data analysis of genomic DNA samples were performed according to the instructions provided by Affymetrix. CytoScan HD chip consisting of CNV and SNP-array probes, which can detect not only CNV but also chimera (chimera ratio >10%) and loss of heterozygosity (LOH), was used. Chromosome analysis suite V3.2 was used to analyze the results, and related databases were used to analyze the CNV properties. CNV can be divided into five categories (14, 15): pathogenic CNV, likely pathogenic CNV, variants of uncertain clinical significance (VUS) CNV, likely benign CNV, and benign CNV. Regarding VUS, SNP-array was recommended for peripheral blood samples of parents, combined with pedigree analysis, to further elucidate the nature of CNV.

### Follow-up of Postnatal Outcomes

All cases were followed-up over telephone to obtain data corresponding to the development of fetuses with ARSA, pregnancy outcome, and postpartum growth and development.

### Statistical Analysis

Microsoft Office Excel 97–2003 was used to input the data and establish the database. SPSS 25.0 software was used for descriptive analysis of the data. The detection rate of pathogenic CNV between both groups was statistically analyzed by the Chi-square test (Fisher’s test), and the difference was considered statistically significant when *P* < 0.05.

## Results

### Association of Aberrant Right Subclavian Artery With Cardiac and Other Ultrasound Abnormalities in Fetuses

The fetuses were divided into two groups: isolated ARSA group and non-isolated ARSA (ARSA with other ultrasound abnormalities) group. Among the 112 fetuses with ARSA, 48 (42.9%, 48/112) were characterized in the isolated ARSA group and 64 (57.1%, 64/112) in the non-isolated ARSA (ARSA combined with other ultrasonic abnormalities) group. In the non-isolated ARSA group, 17 cases (15.2%, 17/112) were associated with congenital heart defects and 47 cases (42.0%, 47/112) were associated with extracardiac abnormalities. Ventricular septal defect (9.8%, 11/112) was the most commonly observed anomaly in fetuses with congenital heart defects, and ultrasonographic soft markers (34.8%, 39/112) were most commonly observed in fetuses with extracardiac abnormalities (Table 1).

**TABLE 1:** Fetuses (64 cases) with non-isolated ARSA.

Classification	Number of fetuses
Combined with congenital heart defects	17
Ventricular septal defect	11
Left aortic arch	3
Pulmonary stenosis	2
Hydropericardium	1
Combined with extracardiac abnormalities	47
Ultrasonographic soft markers	39
Intrauterine growth restriction	1
Central nervous system	1
Acromphalus	1
Urogenital system	2
Polyhydramnios	1
Cleft lip	1
Strephenopodia	1

### Karyotype Analysis

Chromosome karyotype analysis was performed successfully with all 112 samples, and a total of 3 abnormalities (2.7%, 3/112) were detected, including 1 case of trisomy 21, 1 case of trisomy 18, and 1 case of large fragment duplication [46, XY, add(22)(q12)]. Other ultrasound abnormalities were detected in all three fetuses with ARSA and chromosomal abnormalities (Table 2).

**TABLE 2:** Karyotype analysis detected in fetuses with ARSA.

Case	Karyotype analysis	SNP-array	Ultrasonic phenotype	P classification
1	47, XY, +21	arr[hg19] (21) × 3	ARSA, nasal bone dysplasia, the transparent layer of the neck thickened, mild tricuspid regurgitation	P
2	47, XY, +18	arr[hg19] (18) × 3	ARSA, ventricular septal defect, pulmonary artery widening with little pulmonary valve regurgitation	P
3	46, XY, add(22)(q11)	arr[hg19] 22q11.1q11.21(16,888,899-18,649,190) × 4	ARSA, ventricular septal defect	P

*ARSA, aberrant right subclavian artery; P, pathogenic.*

### Single-Nucleotide Polymorphism-Array Results

Single-nucleotide polymorphism-array was performed with 112 samples, and abnormalities were detected in 10 cases (8.9%, 10/112), including 8 cases showing pathogenic CNV and 2 cases of VUS (Tables 2, 3). The eight cases of pathogenic CNV included two cases of aneuploidy, one case of large fragment duplication, two cases of microdeletion, two cases of microduplication, and one case of LOH.

**TABLE 3:** Single-nucleotide polymorphism-array detected in fetuses with ARSA.

Case	SNP-array	Size (Mb)	Ultrasonic phenotype	P classification	Inheritance
1	arr[hg19] 16p11.2(28,786,703-29,032,280) × 3	0.2	ARSA	P	Maternal
2	arr[hg19] 1p36.21p35.2(15,728,288-31,781,279) × 2 hmz, 4p15.2p11(25,981,952-49,063,479) × 2 hmz	16.23	ARSA	P	–
3	arr[hg19] 17p12p11.2(15,759,453-20,547,625) × 3	4.7	ARSA, Strephenopodia	P	–
4	arr[hg19] 22q11.21(18,636,749-21,800,471) × 1	3.16	ARSA, double renal pelvis separation	P	–
5	arr[hg19] 2q13(111,397,196-113,111,856) × 1	1.7	ARSA, ventricular septal defect	P	Maternal
6	arr[hg19] 7q34(139,340,641-139,769,640) × 3	0.4	ARSA	VUS	Maternal
7	arr[hg19] 4q24(106,284,925-107,545,257) × 3	1.2	ARSA, ventricular septal defect	VUS	Maternal

*ARSA, aberrant right subclavian artery; P, pathogenic; VUS, variants of uncertain clinical significance.*

The total detection rate was 7.1% (8/112), as observed using karyotype analysis and SNP-array. The results of the two methods were identical in three cases, including two cases of aneuploidy and one case of large fragment duplication (Table 2). SNP-array also detected five additional cases of pathogenic CNV and two additional cases of VUS. Of the five fetuses demonstrating pathogenic CNV, two showed isolated ARSA, and the other three fetuses showed ARSA associated with other ultrasound abnormalities (Table 3).

### Comparison of Pathogenic Copy Number Variation Between Isolated Aberrant Right Subclavian Artery and Non-isolated Aberrant Right Subclavian Artery Groups

Among the 48 cases in the isolated ARSA group, 2 cases showed pathogenic CNV, with a positive rate of 4.2%. Among the 64 cases in the non-isolated ARSA group, 6 cases showed pathogenic CNV, with a positive rate of 9.4%. Although pathogenic CNV in the non-isolated ARSA group was higher than that in the isolated ARSA group, no statistical significance was observed between the two groups (*P* = 0.463, *P* > 0.05) (Table 4).

**TABLE 4:** Phenotypic characteristics of 112 fetuses with ARSA.

Classification	Number of fetuses	Number of pathogenic CNV
Fetuses with isolated ARSA	48	2 (4.2%)
Fetuses with non-isolated ARSA	64	6 (9.4%)
Total	112	8 (7.1%)

*ARSA, aberrant right subclavian artery; CNV, copy number variation. Fisher’s test, P = 0.463, P > 0.05.*

### Follow-up of Postnatal Outcomes

Of the 112 fetuses with ARSA, successful follow-ups were conducted for 110 fetuses with ARSA; follow-ups for the remaining 2 fetuses were unsuccessful. The parents of eight fetuses with pathogenic CNVs chose to terminate the pregnancies. In addition, four fetuses showed ARSA. Although the results of karyotype analysis and SNP-array were normal, the ultrasound showed severe malformations, and the parents of the fetuses also chose to terminate the pregnancies. The parents of the two fetuses with VUS chose to continue their pregnancies, and the fetuses showed good growth during the postnatal follow-up. The remaining 96 fetuses showing ARSA with normal karyotype analysis and SNP-array all developed well after birth.

## Discussion

With the development of ultrasound technology and improvement in the understanding of fetal ARSA, the prenatal detection rate of ARSA is increasing daily. In this study, 112 fetuses were diagnosed with ARSA by prenatal ultrasound. Trisomy 21 syndrome has been reported in 14–20% of fetuses with ARSA (16, 17). Hence, ARSA is closely related to chromosomal abnormalities. In this study, karyotype analysis and SNP-array were used to detect the genetic etiology of 112 fetuses with ARSA. Chromosomal abnormalities were detected in three cases (2.7%, 3/112) by karyotype analysis. The rate of chromosome abnormalities in this study was significantly lower than those reported in the previous studies (18). However, SNP-array was used to detect five additional cases of pathogenic CNV, including two cases of microdeletion, two cases of microduplication, and one case of LOH. Conventional karyotype analysis can only detect chromosomal fragment abnormalities of over 5–10 MB, while SNP-array can detect low copy number abnormalities of over 10 KB along with normal copy number abnormalities, such as LOH (19, 20). Therefore, SNP-array has advantages in the etiological detection of fetuses with ARSA.

In this study, among 112 fetuses with ARSA, 8 fetuses showed pathogenic CNV, including trisomy 21, trisomy 18, large fragment 22q12 duplication, 22q11.21 microdeletion, 17p12p11.2 microduplication, 16p11.2 microduplication, 2q13 microdeletion, and LOH of 1p36.21p35.2 and 4p15.2p11. The presence of ARSA may increase the risk of trisomy 21 syndrome. In this study, trisomy 21 syndrome was detected in 1 fetus with ARSA, which is consistent with previous studies (9, 18, 21–23). ARSA is also associated with 22q11 deletion syndrome (22q11DS) (24). The 22q11 contains *TBX1*, *SNAP29*, and *CRKL* genes, deletion of which can lead to congenital heart defects and ARSA (25). The manifestation of 22q11DS in patients is varied, primarily including congenital heart defects, thymus hypoplasia, parathyroid dysfunction with hypocalcemia, and developmental delay (25, 26). Moreover, patients with 22q11DS may also demonstrate vascular abnormalities, such as the right aortic arch and ARSA (27–30). One fetus with ARSA was also diagnosed with 22q11DS in this study. The microduplication of 17p12p11.2 comprising the *RAI1* gene can lead to the onset of Potocki–Lupski syndrome (31). The main clinical characteristics of Potocki–Lupski syndrome are abnormal heart development, low intelligence, triangular face, high-zygomatic arch, and palatal dysplasia (32). To date, no studies have reported the relationship between Potocki–Lupski syndrome and ARSA. However, in this study, one fetus with ARSA showed 17p12p11.2 microduplication. Similarly, microduplication of 16p11.2 comprising *ATXN2L*, *TUFM*, *SH2B1*, and *ATP2A1* genes can lead to the onset of microcephaly and autism spectrum disorder (33). No relevant research is available on the relationship between 16p11.2 microduplication and ARSA. The microdeletion of 2q13 comprising *TMEM87B* and *FBLN7* genes can lead to the language delay, abnormal head size, congenital heart defects, and other abnormalities (34). No relevant studies have examined the relationship between 2q13 microdeletion and ARSA. The LOH of 1p36.21p35.2 and 4p15.2p11, which comprise many genes, such as *EFHD2*, *CTRC*, *CELA2A*, *CELA2B*, and *RBPJ*, has also been observed. However, no relevant studies have been published examining the relationship between LOH of 1p36.21p35.2 and 4p15.2p11 and ARSA. The relationship between ARSA and 16p11.2 microduplication, 2q13 microdeletion, and LOH of 1p36.21p35.2 and 4p15.2p11 needs to be analyzed in future studies.

Chaoui et al. (35), Rembouskos et al. (22), and Gul et al. (21) each reported a case of isolated ARSA with trisomy 21. Therefore, some researchers believe that ARSA can be used as an ultrasound soft marker for prenatal screening of fetal chromosome abnormalities, and prenatal chromosome examination should be recommended even if it is diagnosed in isolation. In contrast, other studies do not recommend invasive prenatal testing for fetuses with isolated ARSA unless accompanied by other ultrasound abnormalities (18, 36). The results of our study showed the detection of 16p11.2 microduplication and LOH of 1p36.21p35.2 and 4p15.2p11 in two fetuses with isolated ARSA, respectively. Hence, we suggest that isolated ARSA may also be associated with some microdeletion, microduplication, and LOH, and SNP-array may be necessary for fetuses with isolated ARSA. The rates of pathogenic CNV in the isolated ARSA and non-isolated ARSA groups were 4.2% (2/48) and 9.3% (6/64), respectively. Although the rate of the pathogenic CNV in the non-isolated ARSA group was higher than that in the isolated ARSA group, no statistical significance was observed between the two groups. Therefore, while SNP array should be recommended for fetuses demonstrating non-isolated ARSA, the possibility of fetuses with isolated ARSA being detected with pathogenic CNV should not be ignored.

In addition to being associated with pathogenic CNV, ARSA is also closely related to congenital heart defects (37). Borenstein et al. (6) reported that the incidence of ARSA with congenital heart defects reached 16%. However, due to the small number of cases, the types of congenital heart defects commonly associated with ARSA were not enumerated. In this study, the incidence of ARSA combined with congenital heart defects was slightly lower; 17 fetuses (15.2%, 17/112) were diagnosed with combined congenital heart defects, among which ventricular septal defect was the most common type. The results showed that extracardiac abnormalities were associated with 34.8% (39/112) of fetuses with ARSA. The detection of abnormalities in soft ultrasound markers was the common complication, and the risk of pathogenic CNV increased with other ultrasound abnormalities. In this study, pathogenic CNV was detected in six fetuses showing ARSA combined with other ultrasound abnormalities, including three fetuses with congenital heart defects, two fetuses with abnormal soft ultrasound markers, and one fetus with strephenopodia. Therefore, when prenatal ultrasound detects ARSA in the fetus, it is necessary to observe the fetus carefully for other ultrasound abnormalities.

Variants of uncertain clinical significance has always been controversial, and the detection rate of VUS depends on the type of the microarray chip and the population studied (20, 38). VUS accounts for 1–12% cases in a population (39). Here, the results showed that VUS accounted for 1.8% (2/112) of the total cases, which is consistent with the previous studies. The two fetuses detected with VUS in this study showed good growth and development during postnatal follow-up. In this study, 96 fetuses with ARSA not demonstrating pathogenic CNV showed good growth and development after birth and during follow-up after genetic counseling. However, four fetuses were diagnosed with ARSA combined with other ultrasound abnormalities. Although the genetic analysis was normal, severe ultrasound abnormalities were observed, and the parents chose to terminate the pregnancies. In recent years, next-generation sequencing has been used to detect single-gene mutations and CNV (40–42), which may provide a more comprehensive prenatal genetic diagnosis for fetuses with ARSA and a better evaluation of fetal prognosis.

Our study also has some limitations. First, the sample size was limited, and the results are prone to bias. Second, the methodology was not perfect. Mutations in a single gene can also be the cause underlying ARSA. Therefore, we aim to improve the methodology by adding the whole-exome sequencing analysis in the future. Third, the follow-up data were based only on parental perceptions. In future studies, imaging tests will be added to the follow-up results.

In conclusion, ARSA is a common soft ultrasound marker. Fetuses showing isolated ARSA have a low probability of being detected with pathogenic CNV. However, when ARSA is observed with other ultrasound abnormalities, the risk of pathogenic CNV is increased remarkably. Prenatal genetic counseling and SNP array should be recommended to better assess fetal prognosis.

## Data Availability Statement

The original contributions presented in the study are included in the article/supplementary material, further inquiries can be directed to the corresponding authors.

## Ethics Statement

The studies involving human participants were reviewed and approved by the Ethics Committee of Fujian Maternal and Child Health Hospital. The patients/participants provided their written informed consent to participate in this study.

## Author Contributions

MC wrote the manuscript. HH searched the literature. XQF collected the data. XGF and SX managed the study. LX designed the study. XC interpreted the data. NL revised the manuscript. All authors contributed to the article and approved the submitted version.

## Conflict of Interest

The authors declare that the research was conducted in the absence of any commercial or financial relationships that could be construed as a potential conflict of interest.

## Publisher’s Note

All claims expressed in this article are solely those of the authors and do not necessarily represent those of their affiliated organizations, or those of the publisher, the editors and the reviewers. Any product that may be evaluated in this article, or claim that may be made by its manufacturer, is not guaranteed or endorsed by the publisher.
